# Functional outcomes after laparoscopic versus robotic-assisted rectal resection: a systematic review and meta-analysis

**DOI:** 10.1007/s00464-019-07361-1

**Published:** 2020-02-05

**Authors:** K. F. Kowalewski, L. Seifert, S. Ali, M. W. Schmidt, S. Seide, C. Haney, C. Tapking, A. Shamiyeh, Y. Kulu, T. Hackert, B. P. Müller-Stich, F. Nickel

**Affiliations:** 1 Department of General, Visceral, and Transplantation Surgery, University of Heidelberg, Im Neuenheimer Feld 110, 69120 Heidelberg, Germany; 2 Department of Urology, University Medical Center Mannheim, Heidelberg University, Theodor-Kutzer Ufer 1-3, 68167 Mannheim, Germany; 3 Institute for Medical Biometry and Informatics, Heidelberg University, Im Neuenheimer, Feld 130.3, 69120 Heidelberg, Germany; 4 Klinik für Allgemein-Und Viszeralchirurgie, Kepler Universitätsklinikum GmbH, Med Campus III., Krankenhausstraße 9, 4021 Linz, Austria

**Keywords:** Minimally invasive surgery, Laparoscopy, Robotic-assisted surgery, Rectal cancer, Functional outcomes, Meta-analysis, Evidence-based medicine

## Abstract

Surgical resection is crucial for curative treatment of rectal cancer. Through multidisciplinary treatment, including radiochemotherapy and total mesorectal excision, survival has improved substantially. Consequently, more patients have to deal with side effects of treatment. The most recently introduced surgical technique is robotic-assisted surgery (RAS) which seems equally effective in terms of oncological control compared to laparoscopy. However, RAS enables further advantages which maximize the precision of surgery, thus providing better functional outcomes such as sexual function or contience without compromising oncological results. This review was done according to the PRISMA and AMSTAR-II guidelines and registered with PROSPERO (CRD42018104519). The search was planned with PICO criteria and conducted on Medline, Web of Science and CENTRAL. All screening steps were performed by two independent reviewers. Inclusion criteria were original, comparative studies for laparoscopy vs. RAS for rectal cancer and reporting of functional outcomes. Quality was assessed with the Newcastle–Ottawa scale. The search retrieved 9703 hits, of which 51 studies with 24,319 patients were included. There was a lower rate of urinary retention (non-RCTs: Odds ratio (OR) [95% Confidence Interval (CI)] 0.65 [0.46, 0.92]; RCTs: OR[CI] 1.29[0.08, 21.47]), ileus (non-RCTs: OR[CI] 0.86[0.75, 0.98]; RCTs: OR[CI] 0.80[0.33, 1.93]), less urinary symptoms (non-RCTs mean difference (MD) [CI] − 0.60 [− 1.17, − 0.03]; RCTs: − 1.37 [− 4.18, 1.44]), and higher quality of life for RAS (only non-RCTs: MD[CI]: 2.99 [2.02, 3.95]). No significant differences were found for sexual function (non-RCTs: standardized MD[CI]: 0.46[− 0.13, 1.04]; RCTs: SMD[CI]: 0.09[− 0.14, 0.31]). The current meta-analysis suggests potential benefits for RAS over laparoscopy in terms of functional outcomes after rectal cancer resection. The current evidence is limited due to non-randomized controlled trials and reporting of functional outcomes as secondary endpoints.

**Electronic supplementary material:**

The online version of this article (10.1007/s00464-019-07361-1) contains supplementary material, which is available to authorized users.

## Background

Colorectal Cancer is currently the third most common cancer in the world and the rectum is affected in approximately one-third of cases [1]. The mainstay of curative treatment is surgical resection with lymph node dissection and total or partial mesorectal excision (TME/PME) [2, 3]. Depending on tumor stage localization surgery can be accompanied by (neo)adjuvant (radio)chemotherapy [4].

Surgery can be performed by either traditional open surgery or minimally invasive approaches. International multicenter studies such as the COLOR II trial [5] or COREAN [6, 7] have proven the equivalence of laparoscopy and open surgery for oncological outcomes (e.g., 5-year disease-free survival), while ALaCaRT [8] and ACOSOG [9] showed equivocal results. Additionally, a current meta-analysis confirmed these findings [10]. Furthermore, robotic-assisted surgery (RAS) has been recently implemented in the field of general surgery for performing complex procedures in rectal [11], pancreatic [12] or esophageal surgery [13]. Advantages include 3D vision, a steady camera and the endowrist function with seven degrees of freedom. As for rectal cancer in particular, a recent meta-analysis found no differences between conventional laparoscopy and RAS in terms of oncological outcomes [14]. Additionally, another meta-analysis compared open, RAS, laparoscopy, and transanal TME and found that laparoscopy and RAS enhance postoperative recovery, while open surgery and transanal TME may improve oncological resection [15].

In combination with screening methods, the available treatment options have led to a lower mortality for rectal cancer in the western world over the last decades [16, 17], with an improvement of 5-year survival from 48.1% in the 1970s to now 67.7% [18]. Due to these higher survival rates, there is now a considerably higher number of patients with long-term survival that have to deal with potential side effects of the treatment which can have a profound impact on patient’s quality of life (QoL). These influencing factors include but are not limited to bowel and urinary continence, sexual function as well as the immediate postoperative course.

Even in the established fields of RAS such as urology, there is no clear evidence indicating better oncologic outcomes for RAS when compared to laparoscopic surgery (or open surgery) [19]. However, there is evidence that shows an improvement in functional outcomes for RAS [7, 8]. This might be true for rectal cancer as well. It is frequently hypothesized that the technological advancements of the robotic system might facilitate fine dissection in anatomical planes and could thus improve the intactness of nerves and other structures relevant for functional outcomes. Current studies focus mainly on oncologic outcomes or perioperative parameters [14] or raise considerable concerns for methodologic quality, e.g., without critical appraisal of the quality of evidence or bias analysis [20, 21].

Therefore, the aim of this review is to compare functional outcomes for laparoscopic and RAS for rectal cancer.

## Materials and methods

This systematic review was conducted in line with the Preferred Reporting Items for Systematic Reviews and Meta-Analyses (PRISMA) statement [22] and the AMSTAR 2 criteria [23]. In addition, it was prospectively registered with PROSPERO (CRD42018104519).

### 2.1. ***Search strategy and information sources***

A comprehensive database search including Medline (via PubMed), Web of Science and CENTRAL was performed as suggested by Goossen et al. [24] until November 2018. However, more recent trials which were identified by manual search during the working process were also included if eligible. No restrictions in terms of language, year or study design were applied. Assistance of a librarian from Heidelberg University was sought to optimize the search strategy based on PICO criteria [25]:

**P** (patients)—Male or female patients over the age of 18 with disease of the rectum which requires elective rectal resection.

**I** (intervention)—Robotic-assisted rectal resection.

**C** (comparator)—Conventional laparoscopic rectal resection.

**O** (outcome)—At least one functional outcome.

**S** (Study design)—Comparative studies with all types of study designs.

RCTs and cohort studies were eligible since RCTs are often criticized to not necessarily reflect reality as there are always patient and surgeon specific factors which influence the choice of treatment. However, they were also analyzed separately in order to evaluate whether different study types lead to different outcomes. Furthermore, different types of resection such as AR, ISR, or APR were included. Depending on the study, high and/or low AR were reported together.

### 2.2. ***Screening process and data extraction***

Two reviewers independently screened titles/abstracts and consecutively full-text of eligible studies. Disagreement was solved by consensus or a third reviewer. Data from included studies were extracted into a dedicated data sheet and pre-tested to prove its suitability. In addition, references and grey literature (e.g., abstracts) were searched. In case of similar studies from the same research groups or missing data, authors were contacted to evaluate whether studies report on the same patient clientele and to gather additional data. For example, in order to gain more information from the ROLARR trial [11] for urinary and sexual dysfunction, an official data sharing agreement were signed. In case of multiple publications of one study, the main manuscript was included, but all of the manuscripts were read and included for data extraction in order to gather all available information.

### 2.3. Data items

Data were sought for (1) general information (e.g., year of publication, country), (2) study participant characteristics (e.g., age, disease, BMI) and inclusion/exclusion criteria, (3) type of intervention [laparoscopic vs. robotic-assisted, as well as type of resection (AR, APR, ISR)] and (4) functional outcomes [ileus, urinary retention, international index erectile function (IIEF), international prostate symptom score (IPSS) which includes typical lower urinary tract (frequency, urgency, straining, intermittency, weak stream, nocturia, incomplete emptying), Quality of life scores (QoL)]. Furthermore, analysis was stratified by study design (RCT vs. non-RCT). In case of different follow-up intervals or time periods, the value of the last available follow-up time point was used for analysis.

### 2.4. Inclusion criteria

- Original studies.
- Reporting functional outcomes.
- Comparative studies (robotic vs laparoscopic rectal resection).

### 2.5. Exclusion criteria

- Indication other than rectal cancer.
- No stratified analysis by study arm.
- Studies before 2001 (after the first reported robotic procedure for colorectal cancer) [26].
- Redundant patient population.
- Juvenile studies.
- Cadavers.

### 2.6. Risk of bias in individual studies

Risk of bias in the individual studies was assessed independently by two reviewers with the Newcastle–Ottawa scale (NOS) [27]. Disagreements regarding the risk of bias assessment were resolved with a third party. Publication bias was assessed with the help of funnel plots.

### 2.7. Certainty in evidence

Certainty in evidence was assessed using the GRADE approach [28]. This was done independently by two reviewers using the GRADE Pro Software (McMaster University and Evidence Prime Inc, Ontario, Canada). In case of disagreement a third reviewer was consulted.

### 2.8. Statistical analysis

Statistical analysis was performed by a biometrician (S.S.) at the Department of Medical Biometry and Informatics at Heidelberg University who was otherwise not involved in the study.

Endpoints were either binary or (quasi) continuous. In case of binary data, the odds ratio were used as effect measure and trials were pooled using the Mantel–Haenzel method to account for the sparse number of events [29, 30]. In case of 0 event either trial arm, 0.5 was added as continuity correction to both arms of the trial. The continuous endpoints were mainly estimated by the mean in each trial arm at pre-surgery and different follow-up times. We used the mean difference as effect measure for continuous endpoints, except for the IIEF total score, where trials reported different measures and the standardized mean difference estimated by Hedges’ g was used. If studies reported medians and interquartile ranges, the mean was estimated by the median the standard deviation by the width of the interquartile range divided by 1.35 [31]. For all meta-analyses, random-effects models with the DerSimonian–Laird estimator for between-trial heterogeneity were estimated to account for unexplained variation. Continuous endpoints were pooled using the inverse variance method.

The individual trial results were additionally combined within type of trial as pre-specified subgroups to account for potential bias in the observational trials. Heterogeneity was explored and reported by the *I*^2^-statistic. Funnel plot asymmetry was assessed graphically for all endpoints, however, no test on funnel plot asymmetry was performed as the presence of subgroups on trial type may already have introduced asymmetry.

The analyses were carried out in the software R (version 3.5.1) [32] and its extension meta (version 4.9.2) [33].

### 2.9. Institutional review board

Approval by an internal review board was not applicable since only data which were already published was used for this study.

## Results

### 3.1. Study selection

**Fig. 1:**
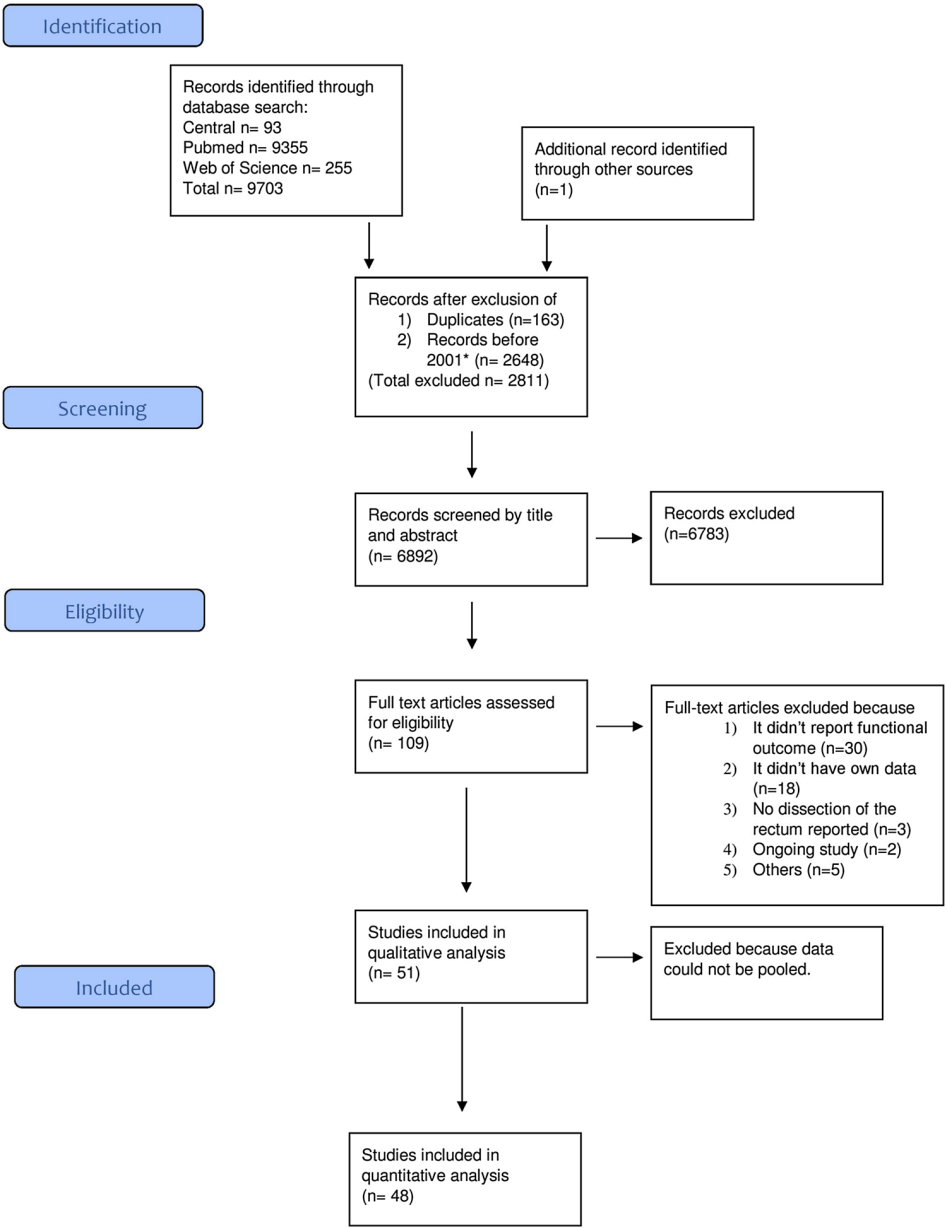
PRISMA flow diagram

A total of 9703 studies were screened for eligibility (see PRISMA flowchart Fig. 1). After abstract and full-text screening 51 studies were included for qualitative and 48 studies for quantitative analyses reporting on 24,319 patients. General information about studies such as year, author, country of origin as well as baseline characteristics are listed in Table 1.

**Table 1:** General information of included studies

First author	Year	Study design	Country	Number of patients		Surgical procedure	Age		Gender (male:female)		BMI		Newcastle Ottawa scale			Total (max:9)
				CL	RAS		CL	RAS	CL	RAS	CL	RAS	Selection	Comparability	Outcome	
Ahmed, J.	2017	Prospective	Portugal	85	99	AR/APR/Hartmann	68^d^ (62–74)	69^d^ (63–75)	58:27	71:28	27^d^ (24–31)	27^d^ (24–30)	★ ★ ✩ ✩	★ ★	★ ★ ✩	6
Allemann, P.	2016	Case matched	Swizerland	40	20	LAR/ISR/APR	65 (13)	64 (12)	24:16	12:8	24,2 (7)	25.9 (9)	★ ★ ★ ✩	★ ★	★ ★ ✩	7
Aselmann, H.	2018	Retrospective	Germany	41	44	NA	65.1 (12)	61.1 (11.45)	24:17	26:18	25.7 (4)	25 (3.8)	★ ★ ★ ✩	★ ★	★ ★ ★	8
Baek, J. H.	2011	Case matched	Korea	41	41	LAR/CA/APR	63.6 (48–87)^c^	63.6 (42–88)^c^	25:16	25:16	25.7 (17.1–34)^c^	26.7 (16.8–40.3)^c^	★ ★ ★ ✩	★ ★	★ ★ ✩	7
Barnajian, M.	2014	Case matched	USA	20	20	LAR/APR	63 (37–82)^a^	62 (44–82)^a^	12:8	12:8	22 (18–31)^a^	22 (18–31)^a^	★ ★ ★ ✩	★ ✩	★ ★ ✩	6
Bedirli, A.	2016	Retrospective	Turkey	28	35	LAR	60.4 (7.1)	64.7 (8.5)	19:9	24:11	23.2 (3.2)	24.7 (3.9)	★ ★ ★ ✩	★ ✩	✩ ✩ ✩	6
Bo, T.	2019	Retrospective	China	1139	556	HAR/LAR/ISR/APR/Hartmann	58 (11.8)	57 (11.9)	708:431	347:209	23 (3.1)	23.3 (3.1)	★ ★ ★ ✩	★ ★	★ ★ ✩	5
Colombo, P. E.	2016	Prospective	France	60	60	ISR	60 (35–85)^a^	62 (34–82)^a^	42:18	40:20	23.8 (17.3–38.6)^a^	25.8 (17.5–41.6)^a^	★ ★ ★ ✩	★ ★	★ ★ ✩	7
D'Annibale, A.	2013	Retrospective	NA	50	50	NA	65.72 (11.6)	66 (12.1)	30:20	30:20	NA (NA)	NA (NA)	★ ★ ★ ★	✩ ✩	★ ★ ★	6
Debakey, Y.	2018	RCT	Egypt	24	21	AR, LAR, uLAR, APR	50.3 (36–64)^e^	53.4 (32–67)^e^	13:11	11:10	Up to 30: 8 over 30: 16	Up to 30: 10 Over 30: 11	★ ★ ★ ★	★ ★	★ ★ ✩	9
Erguner, I.	2013	Prospective	Turkey	37	27	LAR	61.5 (42–80)^a^	54 (24–78)^a^	20:17	14:13	26.75 (19–40)^a^	28.3 (19.8–30.8)^a^	★ ★ ★ ✩	★ ✩	★ ★ ✩	6
Feinberg, A. E.	2016	Retrospective	Canada	8392	472	NA	60.3 (15)	60.1 (15.5)	3965:4427	214:258	28.52 (6.77)	28.22	★ ★ ★ ✩	★ ✩	★ ★ ✩	6
Fernandez, R.	2013	Retrospective	USA	59	13	LAR/APR	64.9 (1.2)	67.9 (2.1)	57:2	13:0	Up to25: 16 25–29.9: 24 over 30: 19	Up to 25: 7 25–29.9: 0 over 30: 6	★ ★ ★ ✩	✩ ✩	★ ★ ✩	5
Feroci, F.	2016	Retrospective	Italy	58	53	NA	66 (33–80)^a^	66 (42–84)^a^	42:16	27:26	24.6 (19–37)^a^	24.6 (18–31)^a^	★ ★ ★ ★	✩ ✩	★ ★ ✩	6
Garfinkle, R.	2018	Retrospective	North America	213	154	LAR, APR	63.8 (13.3)	61.9 (13.5)	127:86	106:48	27.3 (5.8)	28 (6.1)	★ ★ ★ ★	★ ★	★ ★ ✩	8
Gorgun, E.	2016	Retrospective	USA	27	29	APR	60.6 (9.8)	58.8 (10.7)	16:11	22:7	35.2 (5)	34.9 (7.2)	★ ★ ★ ✩	★ ✩	★ ★ ✩	6
Huang, Y. M.	2017	Case matched	Taiwan	38	40	LAR/uLAR/ISR	60.1 (14.2)	60.6 (12.2)	28:10	25:15	24.3 (3.5)	23 (44.4)	★ ★ ★ ✩	★ ✩	★ ★ ✩	6
Ishihara, S.	2018	Retrospective	Japan	100	100	LAR/HAR/ISR/Hartmann	61.3 (NA)	62.1 (NA)	56:44	53:47	22.3 (NA)	22.1 (NA)	★ ★ ★ ✩	★ ★	★ ★ ✩	7
Ielpo, B.	2014	Retrospective	Spain	86	112	LAR/APR/CA	61.6 (11.9)	63.9 (9.5)	67:45	48:38	25.67 (3.36)	26.1 (4.1)	★ ★ ★ ✩	★ ✩	★ ★ ✩	6
Jayne, D.	2017	RCT	10 countries	234	237	HAR/LAR/APR	65.5 (NA)	64.4 (NA)	159:75	161:76	up to 24.9: 87 25–29.9: 92 over 30: 55	up to 24.9: 93 25–29.9: 90 over 30: 54	★ ★ ★ ★	★ ★	★ ★ ★	9
Kamali, D.	2017	Retrospective	UK	18	18	LAR	73 (11)^a^	66 (11)^a^	13:5	11:7	NA (NA)	NA (NA)	★ ★ ★ ✩	★ ✩	★ ★ ✩	6
Kamali, D.	2017	Retrospective	UK	11	11	ELAPE	71 (10.1)	57 (12.7)	NA:NA	NA:NA	NA (NA)	NA (NA)	★ ★ ★ ✩	★ ✩	★ ★ ✩	6
Kang, J.	2013	Case matched	Korea	165	165	SPP/Hartmann/APR	60.4 (11.8)	61.2 (11.4)	97:68	104:61	23.2 (3.1)	23.1 (2.8)	★ ★ ★ ★	★ ✩	★ ★ ★	8
Kim, H. J.	2018	Prospective	Korea	130	130	APR/LAR/CAA	60 (9.3)	60.5 (10.1)	95:35	95:35	23.3 (2.9)	23.7 (3.2)	★ ★ ★ ✩	★ ★	★ ★ ★	8
Kim, J.	2017	Retrospective	Korea	224	224	AR/LAR/ISR/APR	61 (11)	60.7 (11.7)	141:83	145:79	23.4 (3.3)	23.3 (3)	★ ★ ★ ✩	★ ✩	★ ★ ✩	6
Kim, J. C.	2016	Prospective	Korea	486	533	AR/LAR/uLAR/ISR/APR	48 (9)	55 (9)	302:184	333:200	23.8 (3)	24.1 (3.1)	★ ★ ★ ✩	✩ ✩	★ ★ ★	6
Kim, J. Y.	2012	Prospective	Korea	49	30	ISR/Hartmann	56.85^d^ (11.14)	54.13^d^ (8.52)	20:19	18:12	24.01^d^ (2.19)	24.36^d^ (2.44)	★ ★ ★ ★	★ ★	★ ★ ★	9
Kim, M. J.	2018	RCT	South Korea	73	66	LAR/APR	59.7 (NA)	60.4 (NA)	52:21	51:15	23.6 (NA)	24.1 (NA)	★ ★ ★ ★	★ ★	★ ★ ✩	8
Koh, F. H.	2014	Retrospective	Singapore	19	19	uLAR/LAR (TME)	60 (48–75)^a^	62 (47–92)^a^	14:5	15:4	NA (NA)	NA (NA)	★ ★ ★ ✩	✩ ✩	★ ★ ✩	5
Law, W. L.	2017	Retrospective	China	171	220	ISR/Hartmann	67 (NA)^a^	65 (NA)^a^	97:74	148:72	24.6 (NA)^a^	24.9 (NA)^a^	★ ★ ★ ✩	★ ✩	★ ★ ✩	6
Levic, K.	2015	Retrospective	Denmark	36	56	LAR/APR/Hartmann	69 (49–87)^a^	65 (32–83)^a^	17:19	34:22	23.8 (16–32)^a^	24.8 (16–34.5)^a^	★ ★ ★ ✩	★ ✩	★ ★ ★	7
Moghadamyeghaneh, Z.	2015	Retrospective	USA	4777	572	APR	62 (13)	64 (12)	2884:1893	556:316	NA (NA)	NA (NA)	★ ★ ★ ✩	✩ ✩	★ ★ ✩	5
Morelli, L.	2016	Retrospective	Italy	25	50	APR/ISR	68.9 (11.5)	68.8 (10.7)	15:10	33:17	24.3 (4.2)	24.7 (3.5)	★ ★ ★ ✩	★ ✩	★ ★ ✩	6
Panteleimonitis, S.	2017	Retrospective	UK	78	48	AR,APR,Hartmann	70 (63–75.25)^b^	69 (64–74.75)^b^	49:29	35:13	26 (23–32.5)^b^	27 (24.25–29.5)^b^	★ ★ ✩ ✩	★ ✩	★ ★ ✩	6
Panteleimonitis, S.	2018	Case matched	UK	61	63	HAR, LAR, APR, Hartmann	67.25 (NA)	65.8 (NA)	41:20	40:23	32 (30–34)^a^	32 (30–35.7)^a^	★ ★ ✩ ✩	★ ★	★ ★ ✩	5
Park, E. J.	2015	Retrospective	South Korea	84	133	LAR	63.5 (11.2)	59.2 (11.4)	60:24	86:47	23.1 (2.9)	22.9 (2.8)	★ ★ ★ ★	✩ ✩	★ ★ ✩	6
Park, J. S.	2015	Case matched	Korea	106	106	ISR	61.7 (9.6)	59.6 (10.8)	71:35	75:31	23.8 (3.3)	24.3 (2.8)	★ ★ ★ ★	★ ★	★ ★ ✩	8
Park, J. S.	2010	Case matched	Korea	82	48	LAR	63.0 (9.0)	61.2 (9.4)	49:33	24:17	23.2 (3.3)	23.4 (2.6)	★ ★ ★ ✩	★ ★	★ ★ ✩	7
Park, J. S.	2011	Retrospective	Korea	123	52	NA	65.1 (10.3)	57.3 (12.3)	70:53	28:24	26.3 (3.3)	23.7 (2.4)	★ ★ ★ ✩	★ ★	★ ★ ✩	7
Park, S. Y.	2014	Case matched	Korea	32	32	LAR/APR/ISR	Under 60: 15 Over 60: 17	Under 60: 14 over 60: 18	32:0	32:0	23.6 (NA)	23.8 (NA)	★ ★ ★ ✩	★ ✩	★ ★ ✩	6
Park, S. Y.	2013	Retrospective	Korea	40	40	ISR	63.6 (10.6)	57.3 (12.1)	25:15	28:12	24.3 (3.1)	23.9 (2.4)	★ ★ ★ ✩	★ ✩	★ ★ ✩	6
Patriti, A.	2009	RCT	Italy	37	29	LAR/APR	69 (10)	68 (10)	Ratio = 1:2	Ratio = 1:1.6	25.4 (6.44)	24 (6.2)	★ ★ ★ ★	★ ★	★ ★ ✩	7
Rouanet, P.	2018	Retrospective	France	200	200	Transanal/ISR	Under 60: 78^d^ Over 60: 122^d^	Under 60: 78^d^ over 60: 122^d^	136:64	131:69	Up to 30: 170^d^ over 30: 27^d^	Up to 30: 172^d^ Over 30: 28^d^	★ ★ ★ ✩	★ ★	★ ★ ✩	7
Saklani, A. P.	2013	Retrospective	South Korea	64	74	LAR/ISR/CAA	60.1 (10.8)	59.6 (12.3)	46:18	50:24	22.7 (2.9)	23.4 (2.9)	★ ★ ★ ★	★ ✩	★ ★ ✩	7
Serin, K. R.	2015	Retrospective	Turkey	65	14	NA	57 (28–80)^b^	54 (41–71)^b^	65:0	14:0	26 (21–32)^b^	24.7 (23–27)^b^	★ ★ ★ ★	✩ ✩	★ ★ ✩	6
Shiomi, A.	2016	Retrospective	Japan	109	127	LAR/APR/ISR/Hartmann	68 (32–92)^a^	65 (31–87)^a^	65:44	93:34	22.8 (12.8–34.9)^a^	23.7 (17.5–39)^a^	★ ★ ★ ✩	★ ✩	★ ★ ✩	6
Silva-Velazco, J.	2016	Retrospective	USA	118	66	LAR/APR	60 (NA)^a^	59 (NA)^a^	66:52	50:16	27 (NA)^a^	29.5 (NA)^a^	★ ★ ★ ✩	✩ ✩	★ ★ ★	6
Wang, G.	2017	RCT	China	66	71	LAR/Hartmann	58.7 (NA)^c^	60.3 (NA)^c^	66:0	71:0	22.4 (NA)^c^	22.9 (NA)^c^	★ ★ ★ ★	★ ★	★ ★ ✩	8
Yamaguchi, T.	2016	Retrospective	Japan	239	203	LAR/APR/ISR/Hartmann	65.9 (10.8)	64.8 (10.8)	154:85	140:63	23.1 (3.64)	23.4 (3.16)	★ ★ ★ ✩	★ ★	★ ★ ✩	7
Yang, S. X.	2018	Prospective	China	102	91	NA	59.09 (11.01)	59.98 (13.5)	61:41	44:47	22.73 (1.93)	22.98 (1.9)	★ ★ ★ ★	★ ★	★ ✩ ✩	7
Young, M. T.	2014	Retrospective	USA	38	45	LAR	50.1 (18.8)	52.4 (17.2)	16:22	32:13	23.7 (4.6)	28.6 (8.1)	★ ★ ★ ✩	✩ ✩	★ ★ ✩	5

Numbers of BMI and age reported as mean (standard deviation) if not indicated otherwise: ^a^median/range, ^b^median/IQR, ^c^mean/range, ^d^median, ^e^range

*CL* conventional laparoscopy, *RAS* robotic-assisted surgery, *AR* anterior resection, *LAR* low anterior resection, *uLAR* ultralow anterior resection, *HAR* high anterior resection, *APR* abdominoperineal resection, *ISR* intersphincteric resection, *ELAPE* extralevator abdominoperineal, *CAA* coloanal anastomosis excision, *SPP* sphincter-preserving procedure, *TME* total mesorectal excision, *PME* partial mesorectal excision, *BMI* Body Mass Index

### 3.2. Outcomes

#### 3.2.1. Ileus

**Fig. 2:**
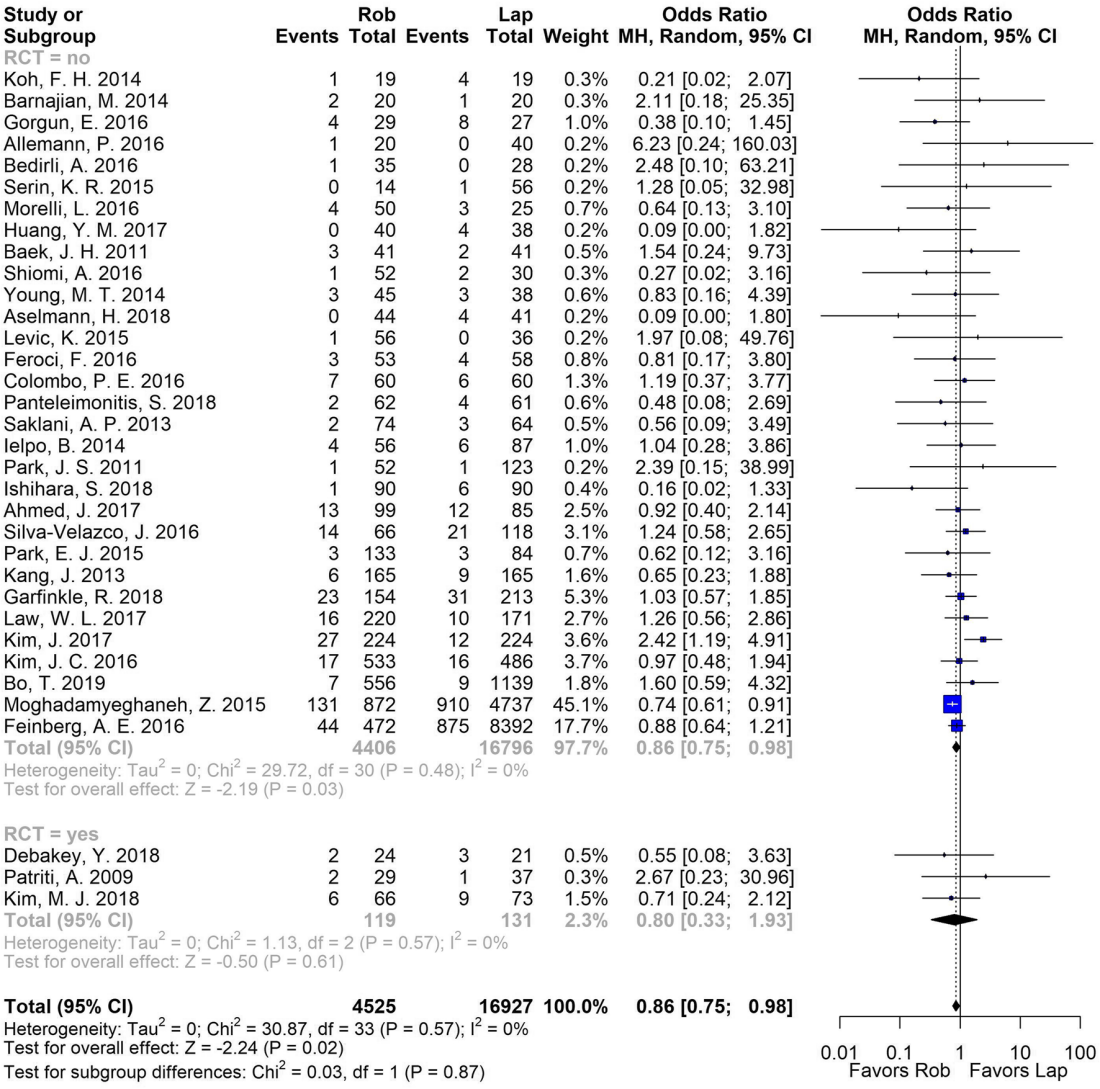
Pooled analysis for ileus

There was a total of 34 studies (31 non-RCTs, 3 RCTs, 21,452 patients) that reported on ileus. There was a significant reduction of the odds of developing an ileus postoperatively in favor of RAS compared to the laparoscopic approach in non-RCTs (odds ratio (OR) [95% confidence interval (CI)] 0.86 [0.75, 0.98]), while there were no differences for RCTs (OR [CI] 0.88 [0.33, 1.93]). The heterogeneity was low in both, overall and subgroup analyses (*I*^2^ = 0%) (Fig. 2).

#### 3.2.2. Urinary retention

**Fig. 3:**
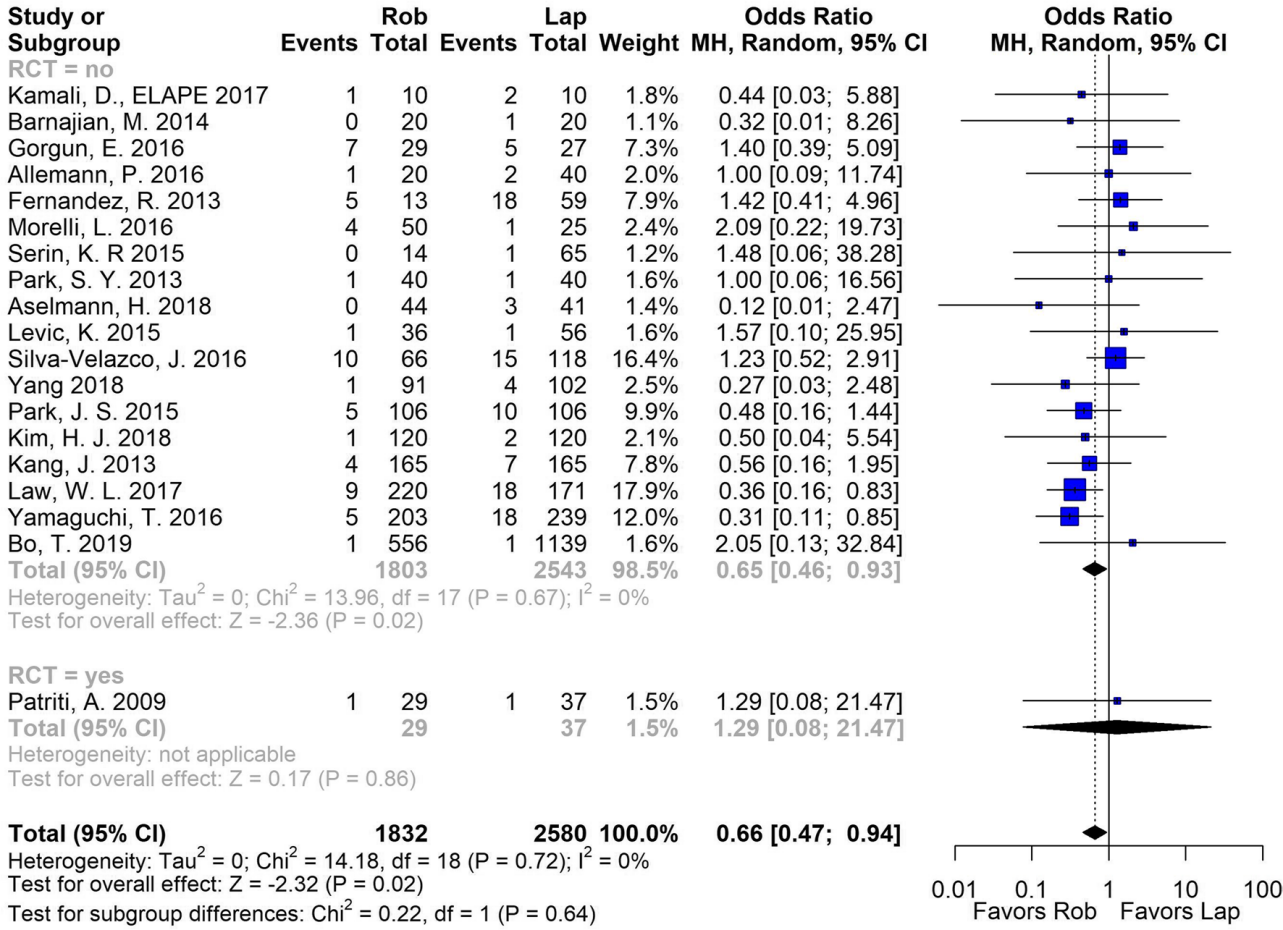
Pooled analysis for urinary retention

Furthermore, for urinary retention (19 non-RCTs, 1 RCT, 4535 patients), there was a significant reduction in urinary retention rate for patients that received RAS compared to laparoscopic in non-RCTs (OR (CI)] 0.65 [0.46, 0.92]). Only one RCT reported on did no show significant differences (OR (CI)] 1.29 [0.08, 21.47]). The heterogeneity was low for both, overall analysis and non-RCTs (*I*^2^ = 0%) (Fig. 3).

#### 3.2.3. Sexual function

**Fig. 4:**
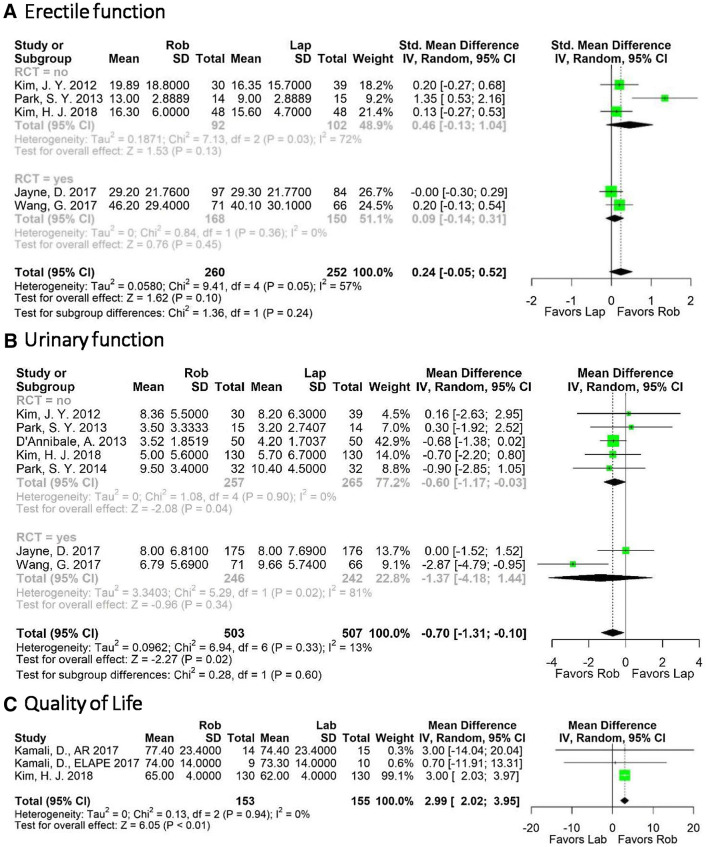
Pooled analysis for **A** erectile function; **B** urinary function; **C** quality of life

Total IIEF scores were reported in five studies (3 non-RCTs, 2 RCTs, 512 patients). There was no difference for RCTs (standardized mean difference (SDM) [CI] 0.09 [− 0.14, 0.31]) or non-RCTs (SMD (CI)] 0.46 [− 0.13, 1.04]) was noted. Heterogeneity was moderate for overall comparison (*I*^2^ = 57%), high for non-RCTs (*I*^2^ = 72%), while it was absent for RCTs (*I*^2^ = 0%) (Fig. 4A).

Additionally, Kim et al. reported sexual functioning with the QLQ-CR 38 and found a better sexual function after 12-month for the RAS group (RAS mean [CI] 35.2 [26.9, 43.5] versus laparoscopy mean [CI] 23.0 [15.7, 30.2], *p* = 0.032) [34]. Furthermore, Jayne et al. assessed female sexual functioning with the Female Sexual Function Index (FSFI, higher scores indicating better function) and found no significant difference between scores for the laparoscopic and the RAS group at 6-month postoperatively (laparoscopy minus RAS: 1.231; CI − 3.54, 6.00; *p* = 0.60) [11]. Rouanet et al. did not find significant differences for the FSFI or IIEF between RAS and laparoscopy but provided graphical results which were not feasible for pooling [35].

#### 3.2.4. ***Urinary symptoms***

In order to assess urinary symptoms, the IPSS was used by a total of 7 studies (5 non-RCTs, 2 RCTs, 1010 patients). The advantage of RAS was statistically significant for non-RCTs (MD [CI] − 0.60 [− 1.17, − 0.03]), while no more differences were found in RCTs (MD [CI] − 1.37 [− 4.18, 1.44]). Accordingly, heterogeneity was high in RCTs (*I*^2^ = 81%), absent in non-RCTs (I^2^ = 0%) and low in their combined analysis [*I*^2^ = 13 (Fig. 4B)].

#### 3.2.5. ***Quality of life***

Three studies (all non-RCTs; 308 patients) reported usable data on quality of life using the core quality of life questionnaire (QLQ-C30). Patients who underwent RAS had a significantly higher score at different points in time after surgery compared to patients who underwent laparoscopy (MD [CI] 2.99 [2.02, 3.95], *p* < 0.001) (Fig. 4C). The heterogeneity was low (*I*^2^ = 0%). In addition, Rouanet et al. also found no differences between the two groups [35]. Kim et al. reported no differences between laparoscopy and RAS for quality of life at 3 weeks, 3 months and 12 months [34].

#### 3.2.6. Quality of evidence and bias assessment

As only four of the included studies were RCTs, all studies were assessed with the NOS in order to ensure comparability. The included studies were of heterogeneous quality (Table 1). Consequently, the certainty in evidence according to GRADE was rated as moderate to very low (Supplementary Material). Funnel plots were used to assess potential publication bias. However, none of the funnel plots raised concerns for publication bias (Supplementary Material—Statistical Analysis).

## Discussion

This systematic review and meta-analysis summarizes the existing evidence in terms of functional outcomes after robotic-assisted or laparoscopic rectal resection for cancer. Overall the results show advantages in favor of the robotic approach regarding ileus, urinary retention, urinary function, and quality of life, while there were no differences concerning sexual functioning.

When compared to open surgery, laparoscopy has shown to provide potential advantages during the perioperative period such as a faster return of bowel function [36] and a shorter hospital stay after rectal cancer surgery [5]. However, no consistent advantages regarding ileus were recorded in RCTs comparing laparoscopy to open surgery [36]. Conversely, the present pooled analysis provides evidence that RAS may help reduce the rate of postoperative ileus compared to CL.

Urinary retention which requires prolonged catheterization was also significantly reduced for RAS compared to CL in the present analysis.

Mid- and long-term outcomes such as QoL and sexual, urinary and bowel functioning are very relevant to patients after the initial postoperative period. Despite the small number of included studies for this outcome, RAS was associated with improved QoL. The most prevalent assessment score used in the included studies was the QLQ C-30 which was developed by the European Organization for Research and Treatment of Cancer (EORTC) and is most commonly used to measure the QoL for cancer patients [37, 38]. Yet, systematic assessment of QoL has been underrepresented in surgical practice and research. In order to capture all facets of a disease and its impact on QoL, “patient reported outcomes” are gaining widespread acceptance. These are an effective approach to comprehensively collecting patients perception of the underlying disease [39]. Therefore, these measurements should be incorporated into the clinical pathway and especially into surgical trials, in order to gain more insight into the impact of different treatment approaches on QoL and to confirm the hypotheses generated by the presented study.

In terms of urinary and erectile functioning it was noticeable that the outcome measures were mainly scores that were not primarily designed to evaluate patients after rectal surgery. The IPSS was first introduced into urology by the American Urological Association in order to assess patient perception of problems derived from benign prostatic hyperplasia [40]. Therefore, it remains questionable whether this score is the optimal choice seeing as symptoms such as straining or weak stream could still be attributed to obstructive symptoms due to an enlarged prostate. Regarding these limitations, studies should check for potential baseline differences in different age groups and stratified randomization based on baseline characteristics should be considered. Additionally, comparability between sexes is not possible as women obviously require different scores.

Similar considerations can be made when assessing sexual function. For example, the ROLARR trial used the IIEF and FSFI to assess sexual function [11]. Despite the high quality of the trial, only 57% of men and 36% of women completed the assessment scores which underlines the low emphasis and neglect of the relevance of functional outcomes in current trials. Additionally, less than 20% of the included studies reported erectile or sexual function at all. Moreover, while potential advantages regarding ileus, urinary retention rates, urinary symptoms as well as QoL for RAS over CL were found, no differences were found for sexual function. In consequence, in the present study it appears that RAS potentially provides better functional outcomes compared to CL.

Seen from a technical perspective, reasons for improved outcomes of RAS may include better visualization due to 3D vision, thus possibly enabling improved detection of risk structures and more accurate dissection due to the stable instrument platform and precise instrumentation. In addition, RAS seems to unfold its potential with more complex procedures. Hence for procedures with high complexity during the reconstruction phase such as pancreaticoduodenectomy or esophagectomy, the laparoscopic approach has failed to gain widespread acceptance due to its technical difficulty. Therefore, these procedures are mainly performed in specialized centers. For example, the recent Dutch Leopard-II study was terminated early due to increased mortality in the laparoscopic group [41]. A subsequent meta-analysis comparing open and laparoscopic pancreaticoduodenectomy including only RCTs did not find any relevant advantage which would support the use of laparoscopy for this procedure [42, 43]. However, the robotic approach to pancreaticoduodenectomy seems to offer a promising alternative to the open procedure with comparable postoperative outcomes and low mortality and should thus be evaluated in RCTs [12].

However, as controversially discussed, the surgeon herself or himself may be a main contributor to the surgical outcome. While there will always be interpersonal differences between surgeons which can hardly be eliminated, each individual has to go through a considerable learning curve. The presence of learning effects is well acknowledged in the surgical community and structured training programs have been introduced. However, it has been shown that previous experience in open or laparoscopic surgery does no translate into better performance with RAS [44]. With this in mind, the learning curve of RAS for rectal cancer takes approximately 20 to 35 cases [45-47]. In comparison, the learning curve of laparoscopic colorectal surgery has been shown to be between 80 and 150 cases depending on the outcome parameter in a systematic review and international multicenter analysis [48]. Of the included studies in this manuscript, a considerable amount reported on their initial experience with RAS without adjusting for learning effects. An excellent example of accounting for surgeon experience is the ROLARR trial by Jayne et al. [11]. Here it was clearly defined that participating surgeons had to have performed at least 30 minimally invasive rectal cancer resections with at least 10 robotic and 10 laparoscopic procedures. As a matter of fact, the ROLARR trial served as real-world example in a consecutive study by Corrigan et al. that accounted for experience of participating surgeons [49]. While the original paper reported an OR of 0.61 (CI 0.31, 1.21; *p* = 0.16) for the primary endpoint (conversion), the study by Corrigan et al. found that patients who were operated by surgeons with the mean experience of all ROLARR surgeons (153 laparoscopic cases; 68 robotic cases), had a lower OR of 0.40 favoring the robotic approach (CI 0.168, 0.953; *p* = 0.039). Furthermore, for surgeons who had performed at least 100 robotic procedures, the odds of conversion were significantly lower with the robotic approach, independent from the number of laparoscopic procedures performed (i.e. 45, 91 or 180 cases). Overall there are learning effects for both approaches, RAS and laparoscopy, while RAS seems to be easier to adopt, thus less cases (and patients) are needed to optimize quality of surgical care.

Finally, since minimally invasive techniques are more often used, an emerging third option besides RAS and traditional laparoscopy is transanal TME (taTME). taTME offers some potential advantages but also seems to have some drawbacks especially in terms of implementation and learning curves. The available studies on this technique showed contradicting results, hence it remains to be further investigated according to IDEAL stages [50, 51]. In accordance with the previous paragraph stringent patient selection, dedicated training, and high case-volume in specialized centers should be integrated to avoid adverse outcomes [52].

### Limitations

The major limitations of the present systematic review and meta-analysis are the heterogeneous reporting measures for the respective studies. For example, there is an original and a simplified version of the IIEF-5 which were both used in different studies. Further limitations include the use of only aggregated data as there was no access to primary data sources and no stratification by surgical approach (e.g., high and low anterior resection, Hartmann’s procedure, intersphincteric resection). For future trials it would thus be desirable to distinguish between the different procedures since this could influence functional outcomes. However, appropriate statistical measures were applied to account for these variations and all analyses were performed by a senior statistician. Additionally, the studies were not powered for functional outcomes as primary endpoints which might reduce the trust in the presented results.

Furthermore, there was no consistent definition of the operative approach in terms of clear differentiation. For example, between LAR and HAR. Both anatomical landmarks, such as above/below the peritoneal flap as well as distances from the anus in centimeter were reported. The limited quality of the included studies itself resulting from mostly non-randomized studies should not be considered as a limitation, since quality assessment is a crucial step of each systematic review which helps to understand drawbacks of recent studies.

### Implications for future research

As discussed previously, the current evidence remains equivocal but this meta-analysis summarizes the existing data, identifies gaps of the current evidence and thus can serve as a basis for generating hypotheses and performing sample size calculations of further studies with functional outcome as primary outcome. In addition, the term functional outcomes must be further defined and clarified. For example, some scores were adopted from other specialties such as urology and might fail to assess further symptoms that are specific to rectal surgery. Along with this, patient characteristics and subgroups (body mass index, gender, previous surgery or neoadjuvant treatment) should be taken into consideration in order to provide patients with a more individualized treatment. Finally, full analysis for the learning curve and differences in tumor stages as well as the different procedures should be further investigated.

## Conclusions

The current systematic review and meta-analysis suggests that there are potential benefits for robotic-assisted surgery over traditional laparoscopy in terms of functional outcomes after rectal cancer resection in non-randomized trials. The current evidence is limited due to a lack of RCTs and the reporting of functional outcomes as secondary endpoints. While robotic-assisted surgery has proven safe with regard to oncological endpoints, there is a need for high-quality randomized-controlled trials which are adequately powered for functional outcomes (e.g., urinary function, sexual function, quality of life).

### Electronic supplementary material

Below is the link to the electronic supplementary material.

Supplementary file1 (PDF 27 kb)

Supplementary file2 (PDF 3425 kb)

Supplementary file3 (PDF 29 kb)

## Appendix

PubMed search strategy

(Rectal Cancer [Title/Abstract] OR Rectum Cancer* [Title/Abstract] OR Rectal Tumor* [Title/Abstract] OR Rectum Neoplasm* [Title/Abstract] OR Total mesorectal exicision [Title/Abstract] OR tme [Title/Abstract] OR rectum [Title/Abstract] OR mesorectal [Title/Abstract] OR Rectal Neoplasms [mesh] OR Rectum/surgery [mesh]).

AND

(laparoscopic [Title/Abstract] OR laporoscopy [Title/Abstract] OR minimal access surgery [Title/Abstract] OR minimally access [Title/Abstract] OR "minimally invasive" [Title/Abstract]OR Laparoscopy [mesh] OR Minimally Invasive Surgical Procedures [mesh]).

AND

(Robotic [Title/Abstract] OR robotic surgery [Title/Abstract] OR robotic-assisted surgery [Title/Abstract] OR robot-assisted surgery [Title/Abstract] OR robotic-assisted [Title/Abstract] OR robotic [Title/Abstract] OR robot-assisted [Title/Abstract] OR robot assisted [Title/Abstract] OR da vinci [Title/Abstract] OR da vinci [Title/Abstract]OR Robotic Surgical Procedures [mesh] OR Surgical Procedures, Operative [mesh] OR Minimally Invasive Surgical Procedures [mesh]).
