# Supplementary material for: Functional outcomes after laparoscopic versus robotic-assisted rectal resection: a systematic review and meta-analysis
Source: Surg Endosc. 2020 Feb 5;35(1):81–95. doi: 10.1007/s00464-019-07361-1 (PMC7746565; doi:10.1007/s00464-019-07361-1)
Supplement: Supplementary file 1 — Supplementary file1 (PDF 27 kb) [file 464_2019_7361_MOESM1_ESM.pdf]

Author(s):
Date:
Question: Robotic-assisted surgery compared to laparoscopic surgery for rectal cancer surgery
Setting:
Bibliography:

Certainty assessment							№ of patients		Effect		Certainty	Importance
№ of studies	Study design	Risk of bias	Inconsistency	Indirectness	Imprecision	Other considerations	Robotic-assisted surgery	laparoscopic surgery	Relative (95% CI)	Absolute (95% CI)		
**Ileus**												
31	observational studies	not serious	not serious	not serious	not serious	none	342/4406 (7.8%)	1970/16796 (11.7%)	**OR 0.86** (0.75 to 0.98)	**15 fewer per 1.000** (from 27 fewer to 2 fewer)	⊕⊕◯◯ LOW	
**Urinary retention**												
19	observational studies	not serious	not serious	not serious	not serious	strong association	56/1844 (3.0%)	112/2625 (4.3%)	**OR 0.65** (0.46 to 0.92)	**15 fewer per 1.000** (from 23 fewer to 3 fewer)	⊕⊕⊕◯ MODERATE	
**Sexual function (assessed with: IEEF)**												
5	observational studies	not serious	not serious	serious [a]	not serious	none	92	102	-	SMD **0.46 SD higher** (0.13 lower to 1.04 higher)	⊕◯◯◯ VERY LOW	
**Urinary symptoms (assessed with: IPSS)**												
7	observational studies	not serious	not serious	serious [a]	not serious	none	257	265	-	MD **0.6 fewer** (1.17 fewer to 0.03 fewer)	⊕◯◯◯ VERY LOW	
**Quality of Life (assessed with: QLQ-C-30)**												
3	observational studies	not serious	not serious	serious [a]	not serious	none	153	155	-	MD **2.99 higher** (2.02 higher to 3.95 higher)	⊕◯◯◯ VERY LOW	

CI: Confidence interval; **OR:** Odds ratio; **SMD:** Standardised mean difference; **MD:** Mean difference

Explanations

a. subjective questionnaire
