# Supplementary material for: Functional outcomes after laparoscopic versus robotic-assisted rectal resection: a systematic review and meta-analysis
Source: Surg Endosc. 2020 Feb 5;35(1):81–95. doi: 10.1007/s00464-019-07361-1 (PMC7746565; doi:10.1007/s00464-019-07361-1)
Supplement: Supplementary file 2 — Supplementary file2 (PDF 3425 kb) [file 464_2019_7361_MOESM2_ESM.pdf]

Meta Analysis of Rectal Cancer Studies

November 11, 2019

Biometrician: Svenja Seide
seide@imbi.uni-heidelberg.de

Contacts: Laura Seifert
lauraseifert96@gmail.com

Karl Kowalewski
karl.kowalewski@googlemail.com

Data source: rectal cancer statistic_neu.xlsx

Data supplied by: Karl Kowalewski

Abstract: [ToDo]

Contents

1 Urinary Retention 3

2 Ileus 5

3 QLQ-Scores 7
3.1 QLQ-C-29 . . . 8
3.2 QLQ-C-30 . . . 8

4 IIEF Total Scores 10

5 IPSS Total Scores 11

6 Session Information 13

```
New names:
* ‘Abdominal pain post op lap‘ -> ‘Abdominal pain post op lap...34‘
* ‘Abdominal pain post op lap (SD)‘ -> ‘Abdominal pain post op lap (SD)...35‘
* ‘Abdominal pain post op rob‘ -> ‘Abdominal pain post op rob...36‘
* ‘Abdominal pain post op rob (SD)‘ -> ‘Abdominal pain post op rob (SD)...37‘
* ‘Abdominal pain post op lap‘ -> ‘Abdominal pain post op lap...80‘
* ... and 3 more problems

New names:
* ‘LAP directly postop (wert)‘ -> ‘LAP directly postop (wert)...18‘
* ‘LAP directly postop (wert)‘ -> ‘LAP directly postop (wert)...19‘
* ‘Postop 3 months rob (wert)‘ -> ‘Postop 3 months rob (wert)...20‘
* ‘Postop 3months rob (Abweichung)‘ -> ‘Postop 3months rob (Abweichung)...21‘
* ‘Postop 3 months rob (wert)‘ -> ‘Postop 3 months rob (wert)...24‘
* ... and 1 more problem
```

1 Urinary Retention

Robotic treatment (experimatal group) compared to laproscopic treatment (control group). Results on the OR-scale (non-logarithmic). In case of 0 events in a study-arm, 0.5 is added to each arm of this study for continuity correction. Assuming that “urinary retention” is an undesirable event, OR $<$ 1 favors Rob, while OR $>$ 1 favors Lab. Studies are sorted by total sample size (from small to large) in the forest plot.

Study or Subgroup	Rob Events	Rob Total	Lap Events	Lap Total	Odds Ratio MH, Random, 95% CI
RCT = no					
Kamali, D., ELAPE 2017	1	10	2	10	0.44 [0.03; 5.88]
Barnajian, M. 2014	0	20	1	20	0.32 [0.01; 8.26]
Gorgun, E. 2016	7	29	5	27	1.40 [0.39; 5.09]
Allemann, P. 2016	1	20	2	40	1.00 [0.09; 11.74]
Fernandez, R. 2013	5	13	18	59	1.42 [0.41; 4.96]
Morelli, L. 2016	4	50	1	25	2.09 [0.22; 19.73]
Serin, K. R 2015	0	14	1	65	1.48 [0.06; 38.28]
Park, S. Y. 2013	1	40	1	40	1.00 [0.06; 16.56]
Aselmann, H. 2018	0	44	3	41	0.12 [0.01; 2.47]
Levic, K. 2015	1	36	1	56	1.57 [0.10; 25.95]
Park, J. S. 2010	0	41	2	82	0.39 [0.02; 8.27]
Silva-Velazco, J. 2016	10	66	15	118	1.23 [0.52; 2.91]
Yang 2018	1	91	4	102	0.27 [0.03; 2.48]
Park, J. S. 2015	5	106	10	106	0.48 [0.16; 1.44]
Kim, H. J. 2018	1	120	2	120	0.50 [0.04; 5.54]
Kang, J. 2013	4	165	7	165	0.56 [0.16; 1.95]
Law, W. L. 2017	9	220	18	171	0.36 [0.16; 0.83]
Yamaguchi, T. 2016	5	203	18	239	0.31 [0.11; 0.85]
Bo, T. 2019	1	556	1	1139	2.05 [0.13; 32.84]
Total (95% CI)		1844		2625	0.65 [0.46; 0.92]
Heterogeneity: $Tau^2 = 0$; $Chi^2 = 14.08$, df = 18 (P = 0.72); $I^2 = 0\%$					
Test for overall effect: Z = -2.42 (P = 0.02)					
RCT = yes					
Patriti, A. 2009	1	29	1	37	1.29 [0.08; 21.47]
Total (95% CI)		29		37	1.29 [0.08; 21.47]
Heterogeneity: not applicable					
Test for overall effect: Z = 0.17 (P = 0.86)					
Heterogeneity: $Tau^2 = 0$; $Chi^2 = 14.30$, df = 19 (P = 0.77); $I^2 = 0\%$					
Test for subgroup differences: $Chi^2 = 0.22$, df = 1 (P = 0.64)					

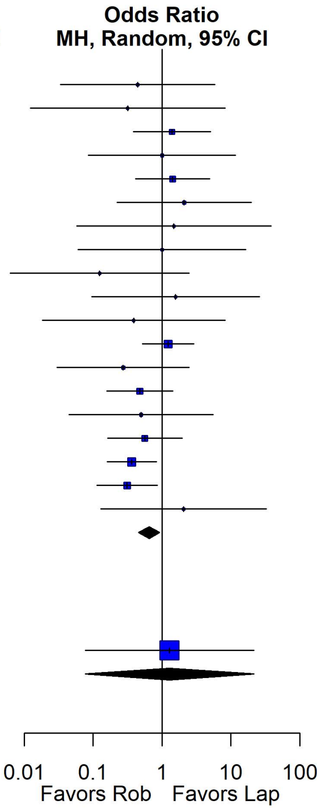

Figure 1: Forest plot for meta-analysis of urinary retention

```
Number of studies combined: k = 20
```

```
                         OR            95%-CI     z p-value
Random effects model 0.6561 [0.4636; 0.9286] -2.38  0.0174

Quantifying heterogeneity:
tau^2 = 0; H = 1.00 [1.00; 1.20]; I^2 = 0.0% [0.0%; 30.8%]

Quantifying residual heterogeneity:
H = 1.00 [1.00; 1.24]; I^2 = 0.0% [0.0%; 34.7%]

Test of heterogeneity:
     Q d.f. p-value
 14.30   19  0.7659

Results for subgroups (random effects model):
            k     OR            95%-CI     Q tau^2  I^2
RCT = no   19 0.6494 [0.4576;  0.9215] 14.08     0 0.0%
RCT = yes   1 1.2857 [0.0770; 21.4725]  0.00    --   --

Test for subgroup differences (random effects model):
                    Q d.f. p-value
Between groups   0.22    1  0.6370

Details on meta-analytical method:
- Mantel-Haenszel method
- DerSimonian-Laird estimator for tau^2
- Continuity correction of 0.5 in studies with zero cell frequencies
```

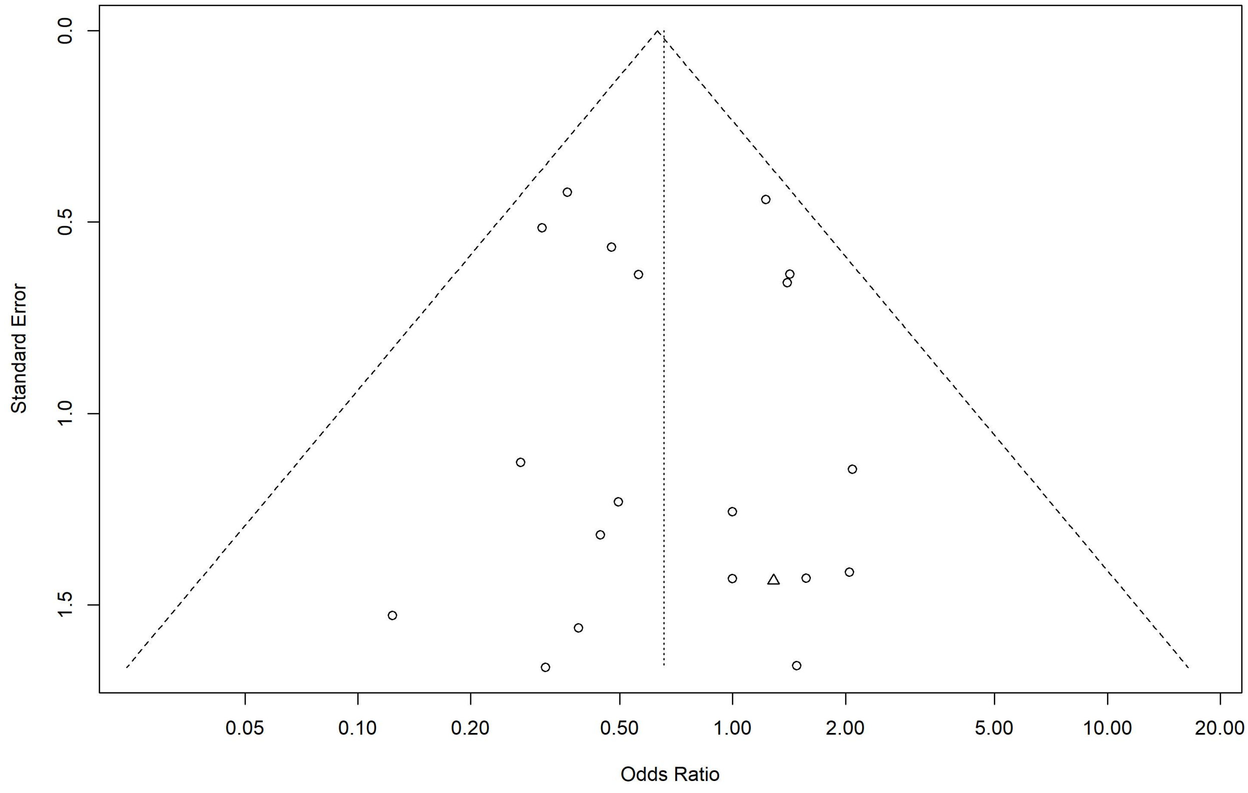

Figure 2: Funnel plot for meta-analysis of urinary retention

2 Ileus

Robotic treatment (experimental group) compared to laproscopic treatment (control group). Results on the OR-scale (non-logarithmic). In case of 0 events in a study-arm, 0.5 is added to each arm of this study for continuity correction. Assuming that "ileus" is an undesirable event, OR $<$ 1 favors Rob, while OR $>$ 1 favors Lab. Studies are sorted by total sample size (from small to large) in the forest plot.

Study or Subgroup	Rob Events	Rob Total	Lap Events	Lap Total	Odds Ratio MH, Random, 95% CI
**RCT = no**					
Koh, F. H. 2014	1	19	4	19	0.21 [0.02; 2.07]
Barnajian, M. 2014	2	20	1	20	2.11 [0.18; 25.35]
Gorgun, E. 2016	4	29	8	27	0.38 [0.10; 1.45]
Allemann, P. 2016	1	20	0	40	6.23 [0.24; 160.03]
Bedirli, A. 2016	1	35	0	28	2.48 [0.10; 63.21]
Serin, K. R. 2015	0	14	1	56	1.28 [0.05; 32.98]
Morelli, L. 2016	4	50	3	25	0.64 [0.13; 3.10]
Huang, Y. M. 2017	0	40	4	38	0.09 [0.00; 1.82]
Baek, J. H. 2011	3	41	2	41	1.54 [0.24; 9.73]
Shiomi, A. 2016	1	52	2	30	0.27 [0.02; 3.16]
Young, M. T. 2014	3	45	3	38	0.83 [0.16; 4.39]
Aselmann, H. 2018	0	44	4	41	0.09 [0.00; 1.80]
Levic, K. 2015	1	56	0	36	1.97 [0.08; 49.76]
Feroci, F. 2016	3	53	4	58	0.81 [0.17; 3.80]
Colombo, P. E. 2016	7	60	6	60	1.19 [0.37; 3.77]
Panteleimonitis, S. 2018	2	62	4	61	0.48 [0.08; 2.69]
Saklani, A. P. 2013	2	74	3	64	0.56 [0.09; 3.49]
Ielpo, B. 2014	4	56	6	87	1.04 [0.28; 3.86]
Park, J. S. 2011	1	52	1	123	2.39 [0.15; 38.99]
Ishihara, S. 2018	1	90	6	90	0.16 [0.02; 1.33]
Ahmed, J. 2017	13	99	12	85	0.92 [0.40; 2.14]
Silva-Velazco, J. 2016	14	66	21	118	1.24 [0.58; 2.65]
Park, E. J. 2015	3	133	3	84	0.62 [0.12; 3.16]
Kang, J. 2013	6	165	9	165	0.65 [0.23; 1.88]
Garfinkle, R. 2018	23	154	31	213	1.03 [0.57; 1.85]
Law, W. L. 2017	16	220	10	171	1.26 [0.56; 2.86]
Kim, J. 2017	27	224	12	224	2.42 [1.19; 4.91]
Kim, J. C. 2016	17	533	16	486	0.97 [0.48; 1.94]
Bo, T. 2019	7	556	9	1139	1.60 [0.59; 4.32]
Moghadamyeghaneh, Z. 2015	131	872	910	4737	0.74 [0.61; 0.91]
Feinberg, A. E. 2016	44	472	875	8392	0.88 [0.64; 1.21]
**Total (95% CI)**		**4406**		**16796**	**0.86 [0.75; 0.98]**
Heterogeneity: Tau^2 = 0; Chi^2 = 29.72, df = 30 (P = 0.48); I^2 = 0%					
Test for overall effect: Z = -2.19 (P = 0.03)					
**RCT = yes**					
Debakey, Y. 2018	2	24	3	21	0.55 [0.08; 3.63]
Patriti, A. 2009	2	29	1	37	2.67 [0.23; 30.96]
Kim, M. J. 2018	6	66	9	73	0.71 [0.24; 2.12]
**Total (95% CI)**		**119**		**131**	**0.80 [0.33; 1.93]**
Heterogeneity: Tau^2 = 0; Chi^2 = 1.13, df = 2 (P = 0.57); I^2 = 0%					
Test for overall effect: Z = -0.50 (P = 0.61)					
Heterogeneity: Tau^2 = 0; Chi^2 = 30.87, df = 33 (P = 0.57); I^2 = 0%					
Test for subgroup differences: Chi^2 = 0.03, df = 1 (P = 0.87)					

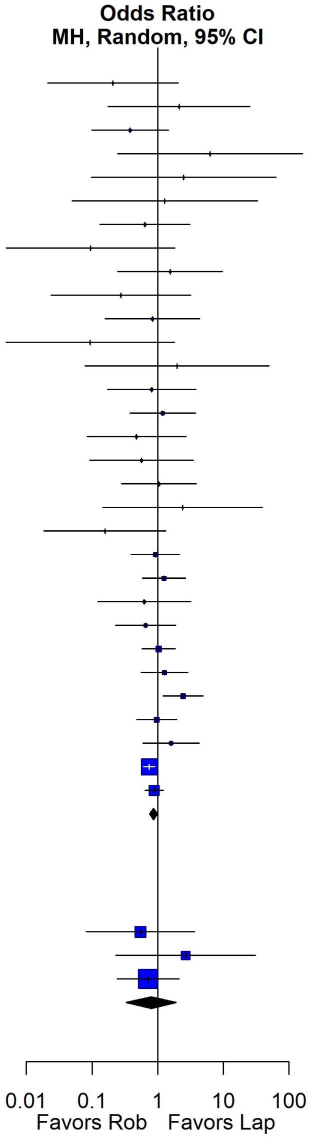

Figure 3: Forest plot for meta-analysis of Ileus

```
Number of studies combined: k = 34
```

```
                         OR            95%-CI     z p-value
Random effects model 0.8578 [0.7503; 0.9807] -2.24  0.0248

Quantifying heterogeneity:
tau^2 = 0; H = 1.00 [1.00; 1.24]; I^2 = 0.0% [0.0%; 34.5%]

Quantifying residual heterogeneity:
H = 1.00 [1.00; 1.26]; I^2 = 0.0% [0.0%; 36.9%]

Test of heterogeneity:
     Q d.f. p-value
 30.87   33  0.5735

Results for subgroups (random effects model):
            k     OR            95%-CI     Q tau^2  I^2
RCT = no   31 0.8593 [0.7504; 0.9840] 29.72     0 0.0%
RCT = yes   3 0.7967 [0.3297; 1.9253]  1.13     0 0.0%

Test for subgroup differences (random effects model):
                    Q d.f. p-value
Between groups   0.03    1  0.8681

Details on meta-analytical method:
- Mantel-Haenszel method
- DerSimonian-Laird estimator for tau^2
- Continuity correction of 0.5 in studies with zero cell frequencies
```

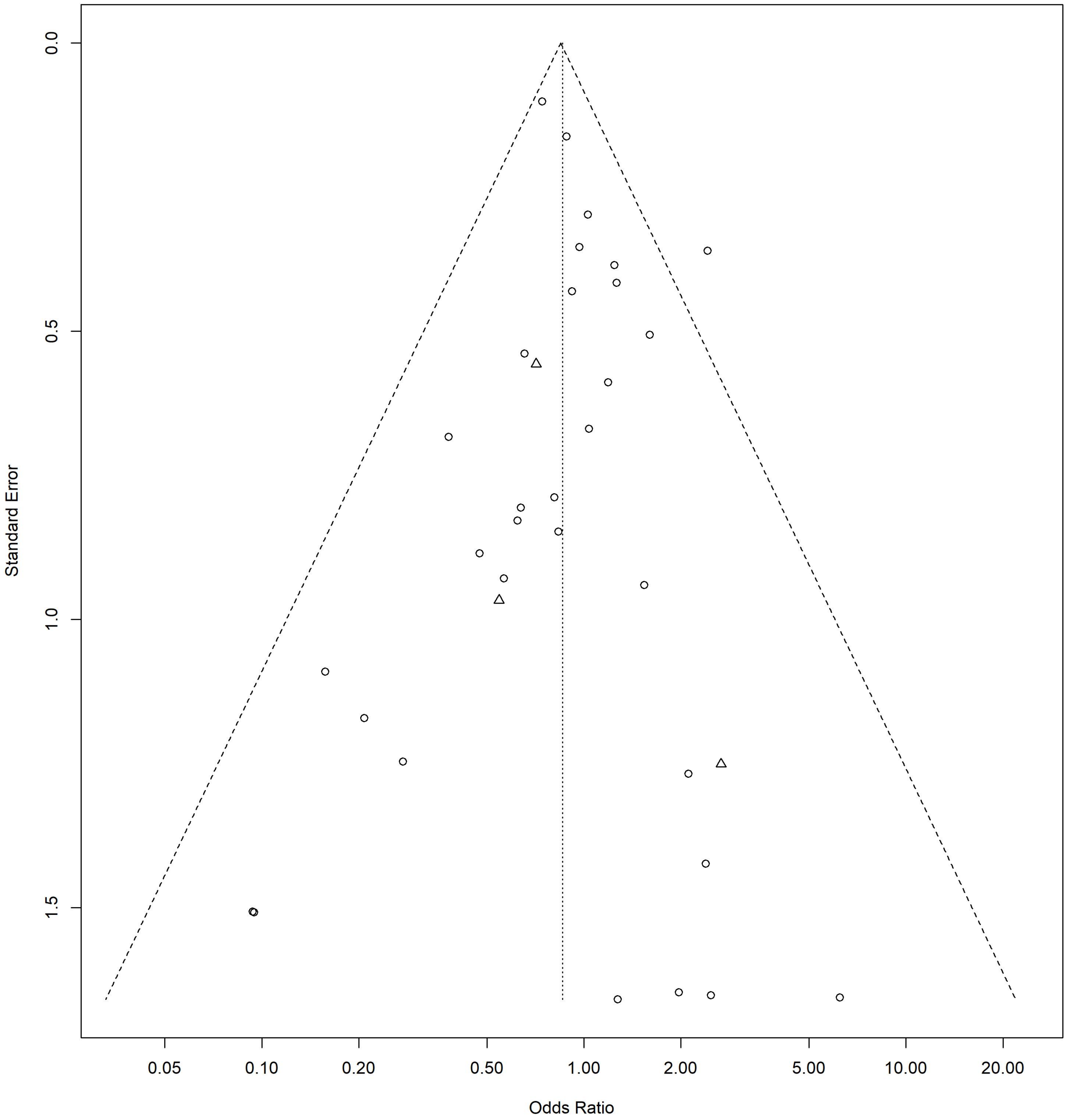

Figure 4: Funnel plot for meta-analysis of Ileus

3 QLQ-Scores

Robotic treatment (experimental group) compared to laproscopic treatment (control group). Results as difference in means. Designations in the forest plot assume a higher mean differences to be better in all cases which translates into a higher score being assumed better.

3.1 QLQ-C-29

Study	Rob Mean	Rob SD	Rob Total	Lab Mean	Lab SD	Lab Total	Mean Difference IV, Random, 95% CI
Kamali, D., ELAPE 2017	74.00	14.0000	9	73.30	14.0000	10	0.70 [-11.91; 13.31]

Heterogeneity: Tau^2 = NA; Chi^2 = 0.00, df = 0 (P = NA); I^2 = NA%

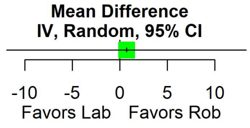

Figure 5: Forest plot for meta-analysis of QLQ-C-29 global health

```
     MD              95%-CI    z p-value
 0.7000 [-11.9076; 13.3076] 0.11  0.9133

Details:
- Inverse variance method
```

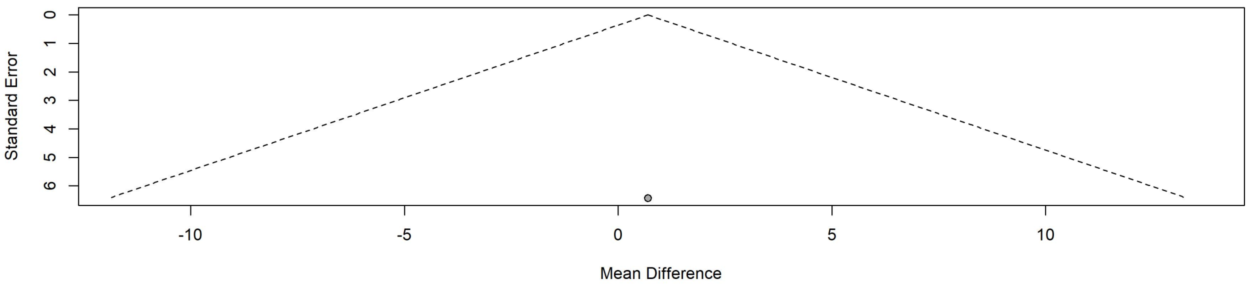

Figure 6: Funnel plot for meta-analysis of QLQ-C-29 global health

3.2 QLQ-C-30

Study	Rob Mean	Rob SD	Rob Total	Lab Mean	Lab SD	Lab Total	Mean Difference IV, Random, 95% CI
Kamali, D., AR 2017	77.40	23.4000	14	74.40	23.4000	15	3.00 [-14.04; 20.04]
Kamali, D., ELAPE 2017	74.00	14.0000	9	73.30	14.0000	10	0.70 [-11.91; 13.31]
Kim, H. J. 2018	65.00	4.0000	130	62.00	4.0000	130	3.00 [2.03; 3.97]

Heterogeneity: Tau^2 = 0; Chi^2 = 0.13, df = 2 (P = 0.94); I^2 = 0%

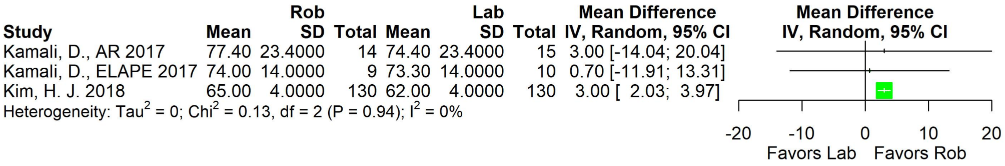

Figure 7: Forest plot for meta-analysis of QLQ-C-30 global health

```
Number of studies combined: k = 3
```

```
                         MD           95%-CI    z  p-value
Random effects model 2.9864 [2.0185; 3.9544] 6.05 < 0.0001

Quantifying heterogeneity:
tau^2 = 0; H = 1.00 [1.00; 1.00]; I^2 = 0.0% [0.0%; 0.0%]

Test of heterogeneity:
    Q d.f. p-value
 0.13    2  0.9384

Details on meta-analytical method:
- Inverse variance method
- DerSimonian-Laird estimator for tau^2
```

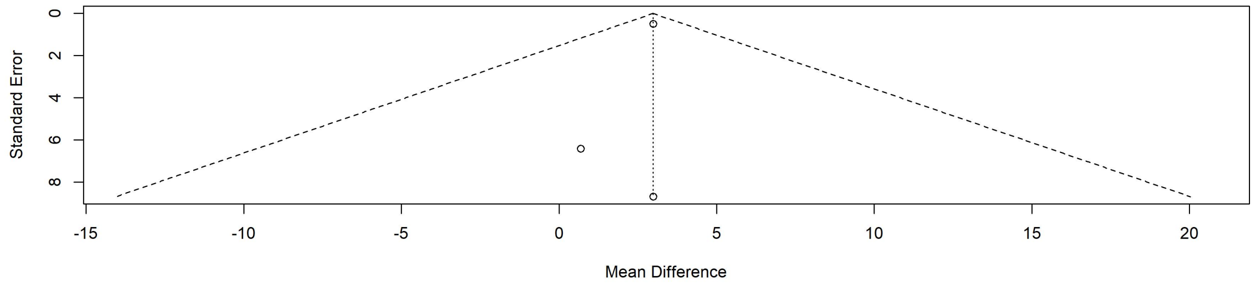

Figure 8: Funnel plot for meta-analysis of QLQ-C-30 global health

4 IIEF Total Scores

Robotic treatment (experimental group) compared to laproscopic treatment (control group). Results as mean differences and assuming a higher score being better. Assuming that the data is assymtotically normal (especially symmetric) in the studies reporting median and range, mean and standard deviation are estimated.

Study or Subgroup	Rob Mean	Rob SD	Rob Total	Lap Mean	Lap SD	Lap Total	Std. Mean Difference IV, Random, 95% CI
RCT = no							
Kim, J. Y. 2012	19.89	18.8000	30	16.35	15.7000	39	0.20 [-0.27; 0.68]
Park, S. Y. 2013	13.00	2.8889	14	9.00	2.8889	15	1.35 [0.53; 2.16]
Kim, H. J. 2018	16.30	6.0000	48	15.60	4.7000	48	0.13 [-0.27; 0.53]
Total (95% CI)			92			102	0.46 [-0.13; 1.04]
Heterogeneity: $Tau^2 = 0.1871$; $Chi^2 = 7.13$, df = 2 (P = 0.03); $I^2 = 72\%$							
Test for overall effect: Z = 1.53 (P = 0.13)							
RCT = yes							
Jayne, D. 2017	29.20	21.7600	97	29.30	21.7700	84	-0.00 [-0.30; 0.29]
Wang, G. 2017	46.20	29.4000	71	40.10	30.1000	66	0.20 [-0.13; 0.54]
Total (95% CI)			168			150	0.09 [-0.14; 0.31]
Heterogeneity: $Tau^2 = 0$; $Chi^2 = 0.84$, df = 1 (P = 0.36); $I^2 = 0\%$							
Test for overall effect: Z = 0.76 (P = 0.45)							
Heterogeneity: $Tau^2 = 0.0580$; $Chi^2 = 9.41$, df = 4 (P = 0.05); $I^2 = 57\%$							
Test for subgroup differences: $Chi^2 = 1.36$, df = 1 (P = 0.24)							

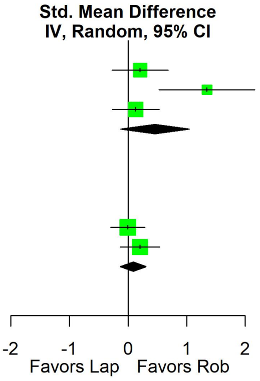

Figure 9: Forest plot for meta-analysis of IIEF at latest time point available, sorted by time of measurement from earliest to latest

```
Number of studies combined: k = 5

                        SMD            95%-CI    z p-value
Random effects model 0.2375 [-0.0491; 0.5242] 1.62  0.1043

Quantifying heterogeneity:
tau^2 = 0.0580; H = 1.53 [1.00; 2.52]; I^2 = 57.5% [0.0%; 84.2%]

Quantifying residual heterogeneity:
H = 1.63 [1.00; 2.81]; I^2 = 62.4% [0.0%; 87.4%]

Test of heterogeneity:
    Q d.f. p-value
 9.41    4  0.0517

Results for subgroups (random effects model):
            k    SMD            95%-CI    Q  tau^2   I^2
RCT = no    3 0.4571 [-0.1287; 1.0429] 7.13 0.1871 72.0%
RCT = yes   2 0.0852 [-0.1353; 0.3057] 0.84      0  0.0%

Test for subgroup differences (random effects model):
                    Q d.f. p-value
Between groups   1.36    1  0.2442

Details on meta-analytical method:
- Inverse variance method
- DerSimonian-Laird estimator for tau^2
- Hedges' g (bias corrected standardised mean difference)
```

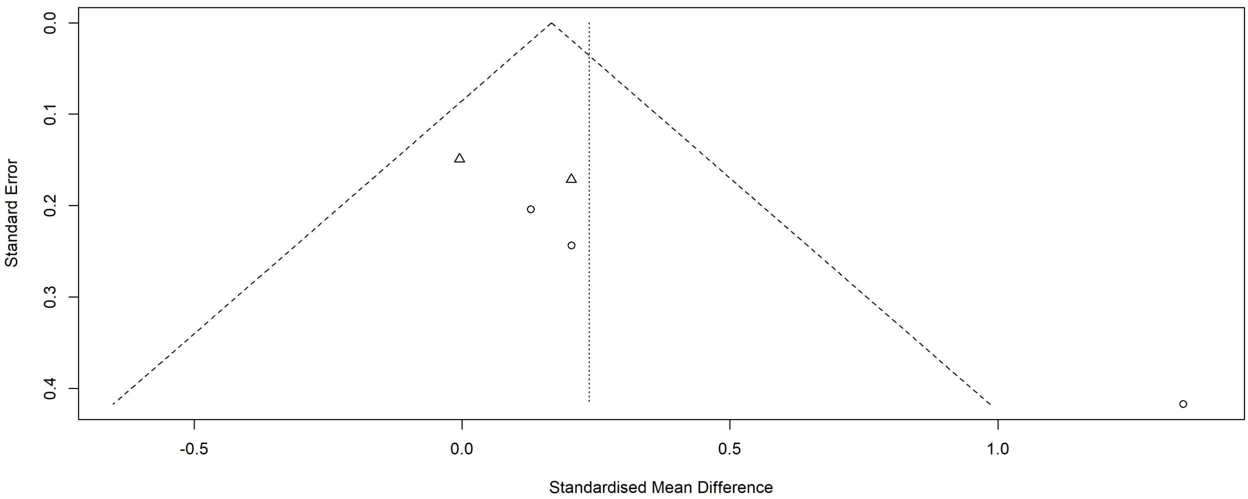

Figure 10: Funnel plot for meta-analysis of IIEF at latest time point available, sorted by time of measurement from earliest to latest

5 IPSS Total Scores

Robotic treatment (experimental group) compared to laproscopic treatment (control group). Results as mean differences and assuming a lower score to be better. Assuming that the data is assymtotically normal (especially symmetric) in the studies reporting median and range, mean and standard deviation are estimated.

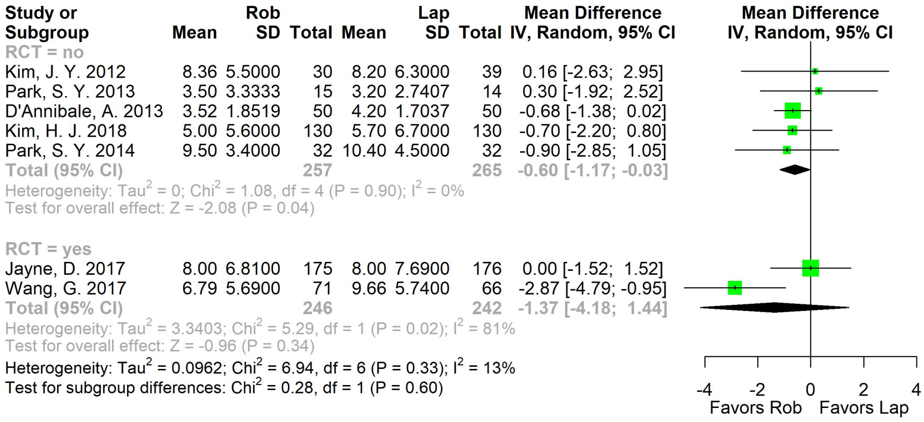

Figure 11: Forest plot for meta-analysis of IPSS at latest time point available, sorted by time of measurement from earliest to latest

```
Number of studies combined: k = 7

                        MD             95%-CI     z p-value
Random effects model -0.7019 [-1.3081; -0.0957] -2.27  0.0232

Quantifying heterogeneity:
```

```
tau^2 = 0.0962; H = 1.08 [1.00; 1.99]; I^2 = 13.5% [0.0%; 74.7%]

Quantifying residual heterogeneity:
H = 1.13 [1.00; 1.71]; I^2 = 21.5% [0.0%; 65.9%]

Test of heterogeneity:
    Q d.f. p-value
 6.94    6  0.3268

Results for subgroups (random effects model):
            k      MD              95%-CI    Q  tau^2   I^2
RCT = no    5 -0.6020 [-1.1706; -0.0335] 1.08      0  0.0%
RCT = yes   2 -1.3733 [-4.1832;  1.4367] 5.29 3.3403 81.1%

Test for subgroup differences (random effects model):
                    Q d.f. p-value
Between groups   0.28    1  0.5980

Details on meta-analytical method:
- Inverse variance method
- DerSimonian-Laird estimator for tau^2
```

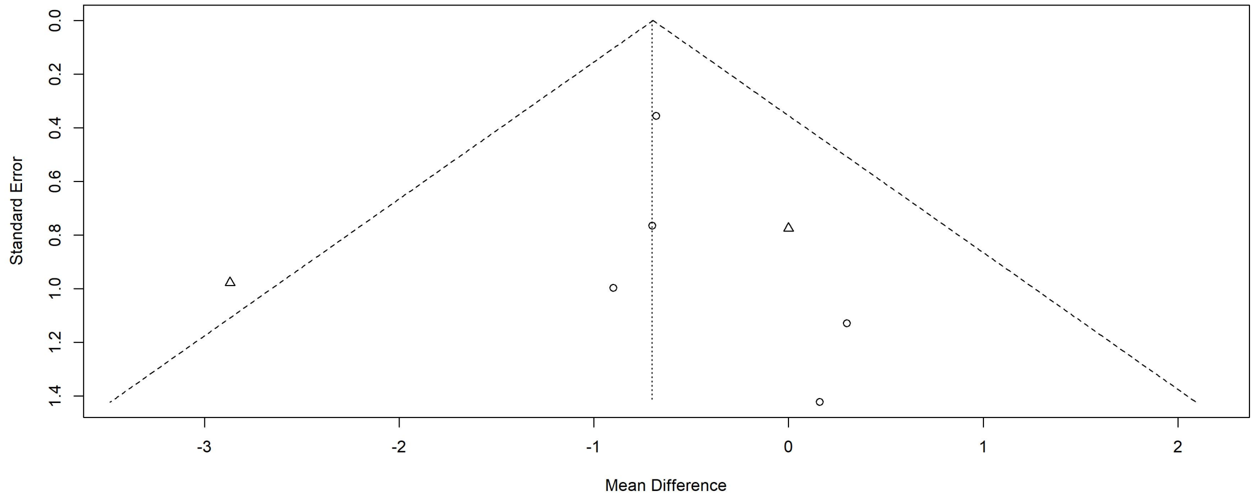

Figure 12: Funnel plot for meta-analysis of IPSS at latest time point available, sorted by time of measurement from earliest to latest

6 Session Information

```
R version 3.5.1 (2018-07-02)
Platform: x86_64-w64-mingw32/x64 (64-bit)
Running under: Windows 10 x64 (build 17134)

Matrix products: default

locale:
[1] LC_COLLATE=German_Germany.1252  LC_CTYPE=German_Germany.1252
[3] LC_MONETARY=German_Germany.1252 LC_NUMERIC=C
[5] LC_TIME=German_Germany.1252

attached base packages:
[1] stats     graphics  grDevices utils     datasets  methods   base

other attached packages:
 [1] readxl_1.1.0    meta_4.9-2      forcats_0.3.0   stringr_1.3.1
 [5] dplyr_0.8.0.1   purrr_0.2.5     readr_1.1.1     tidyr_0.8.2
 [9] tibble_2.1.1    ggplot2_3.1.0   tidyverse_1.2.1

loaded via a namespace (and not attached):
 [1] Rcpp_1.0.0       cellranger_1.1.0 pillar_1.3.1     compiler_3.5.1
 [5] plyr_1.8.4       tools_3.5.1      digest_0.6.18    lubridate_1.7.4
 [9] jsonlite_1.5     evaluate_0.12    nlme_3.1-137     gtable_0.2.0
[13] lattice_0.20-35  pkgconfig_2.0.2  rlang_0.3.4      cli_1.0.1
[17] rstudioapi_0.8   yaml_2.2.0       haven_1.1.2      withr_2.1.2
[21] xml2_1.2.0       httr_1.3.1       knitr_1.20       hms_0.4.2
[25] rprojroot_1.3-2  imbiReport_0.1.0 grid_3.5.1       tidyselect_0.2.5
[29] glue_1.3.0       R6_2.3.0         rmarkdown_1.10   modelr_0.1.2
[33] magrittr_1.5     codetools_0.2-15 backports_1.1.2  scales_1.0.0
[37] htmltools_0.3.6  rvest_0.3.2      assertthat_0.2.0 colorspace_1.3-2
[41] stringi_1.2.4    lazyeval_0.2.1   munsell_0.5.0    broom_0.5.0
[45] crayon_1.3.4
```
