# Supplementary material for: Functional outcomes after laparoscopic versus robotic-assisted rectal resection: a systematic review and meta-analysis
Source: Surg Endosc. 2020 Feb 5;35(1):81–95. doi: 10.1007/s00464-019-07361-1 (PMC7746565; doi:10.1007/s00464-019-07361-1)
Supplement: Supplementary file 3 — Supplementary file3 (PDF 29 kb) [file 464_2019_7361_MOESM3_ESM.pdf]

Summary of findings:

Robotic-assisted surgery compared to laparoscopic surgery for rectal cancer surgery

Patient or population: rectal cancer surgery
Setting:
Intervention: Robotic-assisted surgery
Comparison: laparoscopic surgery

Outcomes	**Anticipated absolute effects*** (95% CI)		Relative effect (95% CI)	№ of participants (studies)	Certainty of the evidence (GRADE)	Comments
	**Risk with laparoscopic surgery**	**Risk with Robotic-assisted surgery**				
Ileus	117 per 1.000	**103 per 1.000** (91 to 115)	**OR 0.86** (0.75 to 0.98)	21202 (31 observational studies)	⨁⨁◯◯ LOW	Lower OR indicates lower rate of ileus retention for robotic-assisted surgery
Urinary retention	43 per 1.000	**28 per 1.000** (20 to 39)	**OR 0.65** (0.46 to 0.92)	4469 (19 observational studies)	⨁⨁⨁◯ MODERATE	Lower OR indicates lower rate of urinary retention for robotic-assisted surgery
Sexual function assessed with: IEEF	-	SMD **0.46 SD higher** (0.13 lower to 1.04 higher)	-	194 (5 observational studies)	⨁◯◯◯ VERY LOW [a]	Higher score indicates better function for robotic-assisted surgery
Urinary symptoms assessed with: IPSS		MD **0.6 fewer** (1.17 fewer to 0.03 fewer)	-	522 (7 observational studies)	⨁◯◯◯ VERY LOW [a]	Lower score indicates less urinary symptoms for robotic-assisted surgery
Quality of Life assessed with: QLQ-C-30		MD **2.99 higher** (2.02 higher to 3.95 higher)	-	308 (3 observational studies)	⨁◯◯◯ VERY LOW [a]	Higher score indicates higher quality of life for robotic-assisted surgery

***The risk in the intervention group** (and its 95% confidence interval) is based on the assumed risk in the comparison group and the **relative effect** of the intervention (and its 95% CI).

CI: Confidence interval; **OR:** Odds ratio; **SMD:** Standardised mean difference; **MD:** Mean difference

GRADE Working Group grades of evidence
High certainty: We are very confident that the true effect lies close to that of the estimate of the effect
Moderate certainty: We are moderately confident in the effect estimate: The true effect is likely to be close to the estimate of the effect, but there is a possibility that it is substantially different
Low certainty: Our confidence in the effect estimate is limited: The true effect may be substantially different from the estimate of the effect
Very low certainty: We have very little confidence in the effect estimate: The true effect is likely to be substantially different from the estimate of effect

Explanations

a. subjective questionnaire
